# Gut microbiota and pre-competitive anxiety in high-performance taekwondo athletes during pre-competition training: an exploratory pilot study

**DOI:** 10.3389/fmicb.2026.1845485

**Published:** 2026-07-07

**Authors:** Lihui Xie, Xuelian Chen, Xinyi Wan, Bing Li

**Affiliations:** School of Physical Education, Southwest University, Chongqing, China

**Keywords:** college, competition anxiety, gut microbiota, high-performance taekwondo athletes, pre-competition training

## Abstract

**Objective:**

Gut microbiota has been implicated in exercise adaptation and emotional regulation, but evidence from high-performance athletes during closed pre-competition training remains limited. This exploratory pilot study examined changes in gut microbiota and competition anxiety across an 8-week pre-competition training period in high-performance taekwondo athletes and explored their associations.

**Methods:**

Eleven high-performance taekwondo athletes (6 men and 5 women) preparing for the 31st FISU World University Games were assessed before training and again at week 8. Stool samples were analyzed using 16S rRNA V3-V4 sequencing, and competition anxiety was measured with the Competitive State Anxiety Inventory-2 (CSAI-2). Given the small single-group repeated-measures design, all microbiome analyses were treated as exploratory. Paired Wilcoxon signed-rank tests were used for displayed pre-post comparisons, and PERMANOVA was applied to the reconstructed beta-diversity coordinate set.

**Results:**

Shannon alpha diversity showed no clear pre-post difference (Wilcoxon *p* = 0.278), and the reconstructed PCoA coordinate set showed no significant overall separation (PERMANOVA R2 = 0.028, *p* = 0.647). Exploratory differential-abundance screening highlighted several candidate taxa, with the strongest signals in the reconstructed dataset observed for Lactobacillus, Enterococcus, norank_f__Muribaculaceae, and Bosea. According to the archived CSAI-2 analysis, cognitive anxiety and somatic anxiety were higher after the preparation period, whereas state self-confidence did not differ significantly. Additional taxa-anxiety associations were observed in the originally reported association subset, but these should be interpreted as nominal exploratory findings only.

**Conclusion:**

In this small single-group pilot cohort, the pre-competition period was accompanied by shifts in selected gut microbiota features and by higher pre-competition anxiety-related responses, particularly in the cognitive and somatic domains.

## 1 Introduction

Taekwondo is one of China’s strongest medal-producing sports at the Summer Olympic Games, yet relatively few studies have examined the integrated physiological and psychological monitoring of high-performance taekwondo athletes during pre-competition training. Traditional monitoring approaches have relied mainly on physiological and biochemical indicators such as blood lactate, hemoglobin, and heart rate (Yao et al., 2017). In recent years, omics-based approaches have increasingly been applied to athlete performance monitoring (Ma et al., 2015; Halson, 2014; Han et al., 2015). Among these, the gut microbiota has emerged as an important area of interest in sports science. The relationship between exercise and the gut microbiota is bidirectional. Exercise may promote potentially beneficial taxa, reduce taxa potentially associated with unfavorable host responses (Munukka et al., 2018), and influence gut microbiota diversity (Denou et al., 2016; Evans et al., 2014). In addition, microbial metabolites such as acetate may contribute to exercise capacity and metabolic adaptation (Scheiman et al., 2019).

Anxiety is a negative emotional state characterized by tension, worry, and heightened arousal (Guo et al., 1989). In sport psychology, competition anxiety refers to the emotional response that occurs when athletes face potentially threatening competitive situations. It is commonly conceptualized in three dimensions: cognitive anxiety, somatic anxiety, and state self-confidence (Weinberg, 1990). Zhang (2000) described competition anxiety as a state of tension, worry, and fear experienced when athletes confront demanding competitive circumstances. Competition anxiety may affect physiological arousal, increase self-doubt, and intensify fear of adverse performance outcomes (Ai et al., 2019). As the technical and tactical standard of high-performance taekwondo athletes continues to improve, competition in this sport has become increasingly demanding, and athletes’ psychological states, especially competition anxiety, may play an important role in performance outcomes.

The gut microbiota-gut-brain axis has become an important research topic in sports science. The gut microbiota is involved in brain development, neural function, and behavior through complex microbiota-gut-brain signaling pathways (Borre et al., 2014; Collins et al., 2012). It can communicate with the brain through neurotransmitters, cytokines, hormones, and other bioactive mediators, thereby influencing mood and behavior (Johannesson et al., 2011). Neurohormonal responses to stress and anxiety are associated with gut microbiota composition, and gut microbial disturbance has been linked to impaired memory and hippocampal cognitive function (Mahony et al., 2015). For example, gut bacteria can convert the dietary amino acid tyrosine into 4-ethyl phenyl sulfate (4EPS), which crosses the blood–brain barrier and interferes with oligodendrocyte maturation, thereby contributing to anxiety-related behavior (Needham et al., 2022). Previous studies have shown that the gut microbiota of elite divers is associated with anxiety and choking under high-pressure training conditions. However, studies examining gut microbiota and competition anxiety in high-performance athletes remain limited. We therefore hypothesized that gut microbiota composition may be associated with changes in competition anxiety in this population.

Therefore, this exploratory pilot study investigated whether gut microbiota composition and competition anxiety changed across an 8-week pre-competition training period in high-performance taekwondo athletes and whether exploratory associations between microbial taxa and CSAI-2 subscale scores could be observed.

## 2 Participants and methods

### 2.1 Participants

This exploratory pilot study enrolled 11 high-performance taekwondo athletes preparing for the 31st FISU World University Games. All participants were national-level competitors training within the same structured pre-competition camp. Baseline demographic and anthropometric characteristics are presented in Table 1. Repeated measurements of gut microbiota, competition anxiety, and anthropometric variables were collected before and after the 8-week pre-competition preparation period. The mixed-sex composition reflected the composition of the available training squad. However, the cohort was too small to support statistically meaningful sex-stratified analyses. Because the athletes were drawn from a small and relatively identifiable pre-competition cohort, additional individual-level information, such as specific weight categories, is not reported in order to preserve participant confidentiality. The study was approved by the Ethics Committee of the School of Physical Education, Southwest University, before participant recruitment and baseline assessment. Written informed consent was obtained from all participants prior to enrollment.

**Table 1:** Basic characteristics of the high-performance taekwondo athlete cohort.

Group	Age (year)	Height (cm)	Weight (kg)	Training years	Sport level	
					Master sportsman	National-level athletes
Male (*n* = 6)	21.3 ± 1.9	180.2 ± 5.7	67.8 ± 5.1	9.8 ± 2.7	1	5
Female (*n* = 5)	20.3 ± 1.5	164.3 ± 5.6	53.6 ± 7.6	8.7 ± 3.1	1	4
Total (*n* = 11)	20.8 ± 1.8	172.3 ± 9.8	60.7 ± 9.6	9.3 ± 2.9	2	9

### 2.2 Methods

#### 2.2.1 Study design and training schedule

Baseline stool samples and anxiety questionnaires were collected before the start of the preparation period. All athletes then completed an 8-week pre-competition training program, with two daily sessions lasting approximately 3 h each in the morning and afternoon. The weekly training schedule is summarized in Table 2, and the overall structure of the 8-week program remained broadly similar across weeks. Follow-up stool samples and anxiety questionnaires were collected in the early morning at the end of week 8. Data collection took place from February to March 2021, before the taekwondo trials for the 31st FISU World University Games.

**Table 2:** Weekly training program during the 8-week preparation period.

Day	Morning (09:00–12:00)	Afternoon (15:00–18:00)
Monday	Technical precision and core stability Dynamic warm-up (20 min) Single-technique target drills: front-leg axe kick, back kick, roundhouse kick on paddle targets and electronic body protectors (40 min) Combination drills: 2–3 kicks with step variations (30 min) Core stability circuit: 4 × (front plank 45 s, side plank 30 s/side, hollow hold 30 s, Russian twist 20 reps)	Maximal strength and core power General warm-up + activation (10 min) Back squat 4 × 5 (80–85% 1RM) Bench press 4 × 5 Deadlift 3 × 5 Bulgarian split squat 3 × 8/leg Ankle/wrist prehabilitation (banded dorsiflexion, wrist curls 3 × 12) Core power: medicine ball rotational throws 4 × 8/side
Tuesday	Tactical drills and reactive agility Warm-up + footwork ladder (15 min) Offensive-defensive transition: call-response partner drill (30 min) Counter-attack scenarios: defend roundhouse → back kick or punch-kick combos (30 min) Core endurance: 3 × (knee-to-elbow plank 30 s, leg raises 15 reps, bicycle crunches 20/side)	Simulated combat and tactical review Warm-up + light sparring prep 3-round conditional sparring (2 min × 3, 60 s rest) with specific constraints (e.g., only front-leg attacks) Video analysis & coach feedback (20 min) Defensive drills: slide-back counter, clinch escape (15 min) Core: isometric holds (3 × 45 s hollow body, arch body)
Wednesday	Technical-tactical combination (volume emphasis) Warm-up + rapid footwork drills Repeated offensive combinations: close-distance, clinch entry, punch-kick transition (40 min) Counter-attack after opponent’s cut-kick (20 min) Core anti-rotation: 3 × 30 s/side Pallof press, 3 × 10 cable woodchops	High-intensity sparring and tactical endurance Warm-up + progressive sparring 4-round sparring (2 min, 45 s rest), official rules, electronic scoring Active recovery: footwork without contact (10 min) Defensive drill: partner pressure, ring control Core: stability ball rollouts 3 × 20
Thursday	Rest	Endurance interval training
Friday	Technical-tactical combination (attack emphasis) Warm-up + reaction time drills Offensive combinations: close-distance, clinch entry, punch-kick transition (30 min) Counter-attack after opponent’s cut-kick (20 min) Core stability: anti-rotation holds 3 × 30 s/side, Pallof press 3 × 10	Simulated combat and defensive drills Same structure as Tuesday afternoon with adjusted constraints (e.g., must initiate attack in first 5 s) Defensive drill: block-counter in ring Core: hanging leg raises 3 × 12
Saturday	Tactical rehearsal and pre-competition simulation Warm-up + footwork under fatigue Full competition simulation: 3 rounds × 2 min, official rules, electronic scoring, referee present (recorded for analysis) Core: 3 × (stir-the-pot 10 reps, ab wheel rollout 8 reps)	Light technique and tactical review Video review of morning simulation Corrective technical practice on identified weaknesses Light reaction drills, no contact Core: mobility-based core (cat-cow, bird-dog) and stretching
Sunday	Rest	Long slow distance (active recovery) 8–10 km run at 60–70% HRmax (conversation pace) Full-body stretching and foam rolling Hydration and nutrition refocus

To minimize major sources of potential confounding, a standardized screening questionnaire was administered before enrollment. Athletes were excluded if they reported systemic antibiotic use within the preceding 3 months, regular probiotic or prebiotic supplementation (> = 3 times per week) within the preceding 4 weeks, diagnosed gastrointestinal disease or prior gastrointestinal surgery, acute illness at enrollment or within the preceding 2 weeks, current use of medications known to affect gut microbiota, or diagnosed psychiatric disorders or anxiolytic/antidepressant medication use. During the study, the athletes trained under COVID-19 closed-campus management and consumed their main meals in the school canteen, which may have reduced some between-individual variability. However, no formal dietary intake assessment was performed, and several potentially relevant variables were not systematically recorded, including habitual dietary intake, gastrointestinal symptoms, alcohol use, sleep quality and duration, menstrual-cycle status, injury status and related medication use, body-mass fluctuation, weight-control practices, and detailed day-to-day training-load variation. These conditions should therefore not be interpreted as full control of dietary or lifestyle confounders.

#### 2.2.2 Gut microbiota assessment

Fecal sample collection and DNA extraction: Stool samples were collected using sterile fecal sampling tubes and stored at −80 C until analysis. DNA extraction was performed with the Rapid DNA Fecal Mini Kit (Qiagen, California, USA) according to the manufacturer’s protocol. DNA concentration was measured with a NanoDrop 2000 instrument (Thermo Scientific, USA).

High-throughput sequencing: Illumina MiSeq sequencing was used to profile bacterial communities in fecal samples. The V3-V4 region of the 16S rRNA gene was amplified, purified, quantified, normalized, and used for paired-end library construction before sequencing. Operational taxonomic unit clustering: Non-redundant sequences were clustered into operational taxonomic units (OTUs) at 97% sequence similarity.

Alpha diversity: Within-sample diversity was summarized with the Shannon index.

Community composition analysis: Principal coordinates analysis (PCoA) based on weighted UniFrac distance was used as a descriptive visualization of beta diversity. For the reconstructed coordinate set used in the revised analysis, exploratory beta-diversity significance was additionally assessed by PERMANOVA. Taxa-level comparisons and correlation analyses are presented as exploratory screening results.

#### 2.2.3 Competition anxiety assessment

Competition anxiety was assessed before and after the preparation period using the Competitive State Anxiety Inventory-2 (CSAI-2). Across the two time points, 22 questionnaire records were collected and all were valid. The CSAI-2, developed by Martens and colleagues, contains 27 items distributed across three subscales: cognitive anxiety, somatic anxiety, and state self-confidence (Weinberg, 1990). Each subscale ranges from 9 to 36, with higher scores indicating higher levels of the corresponding construct. In the present study, the term competition anxiety is used as an umbrella construct, whereas the three CSAI-2 subscales are reported separately when referring to specific questionnaire outcomes.

#### 2.2.4 Statistical analysis

SPSS 26.0 was used for questionnaire analyses. Because of the small sample size and the repeated-measures design, the displayed pre-post comparisons of competition anxiety and Shannon alpha diversity in the reconstructed dataset were evaluated with paired Wilcoxon signed-rank tests and are reported as nominal values.

For gut microbiota analyses, alpha diversity was calculated with Mothur software. Because this pilot study used repeated measurements in a very small sample, all microbiome findings are treated as exploratory rather than confirmatory. Shannon diversity is summarized descriptively, PCoA is used as a descriptive visualization of beta diversity, and exploratory PERMANOVA was applied to the reconstructed coordinate set.

To address the multiple-comparison burden in the reported microbiota summaries available in the revised dataset, Benjamini-Hochberg false discovery rate correction was applied to the reported differential taxa and to the reported taxa-anxiety association set in Table 3. Both unadjusted *p*-values and FDR-adjusted q-values are presented where applicable.

**Table 3:** Reported exploratory Spearman correlations between selected gut microbiota taxa and CSAI-2 subscale scores.

Outcome	Gut microbiota taxon	Correlation coefficient	Unadjusted *p*-value
Cognitive anxiety	*Burkholderia*ceae	0.652	0.041
	*Lachnospira*ceae	−0.451	0.035
Somatic anxiety	Lactobacillaceae	−0.689	0.027
	*Lactobacillus*	−0.689	0.027
State self-confidence	*Faecalibacterium*	0.652	0.041
	*Prevotella*ceae	−0.803	0.005
	*Prevotella*	−0.648	0.043
	*Alloprevotella*	−0.704	0.023
	*Ruminococcus*	−0.751	0.012

## 3 Results

### 3.1 Gut microbiota differences before and after the pre-competition training

#### 3.1.1 Alpha diversity before and after the pre-competition training

**Figure 1:**
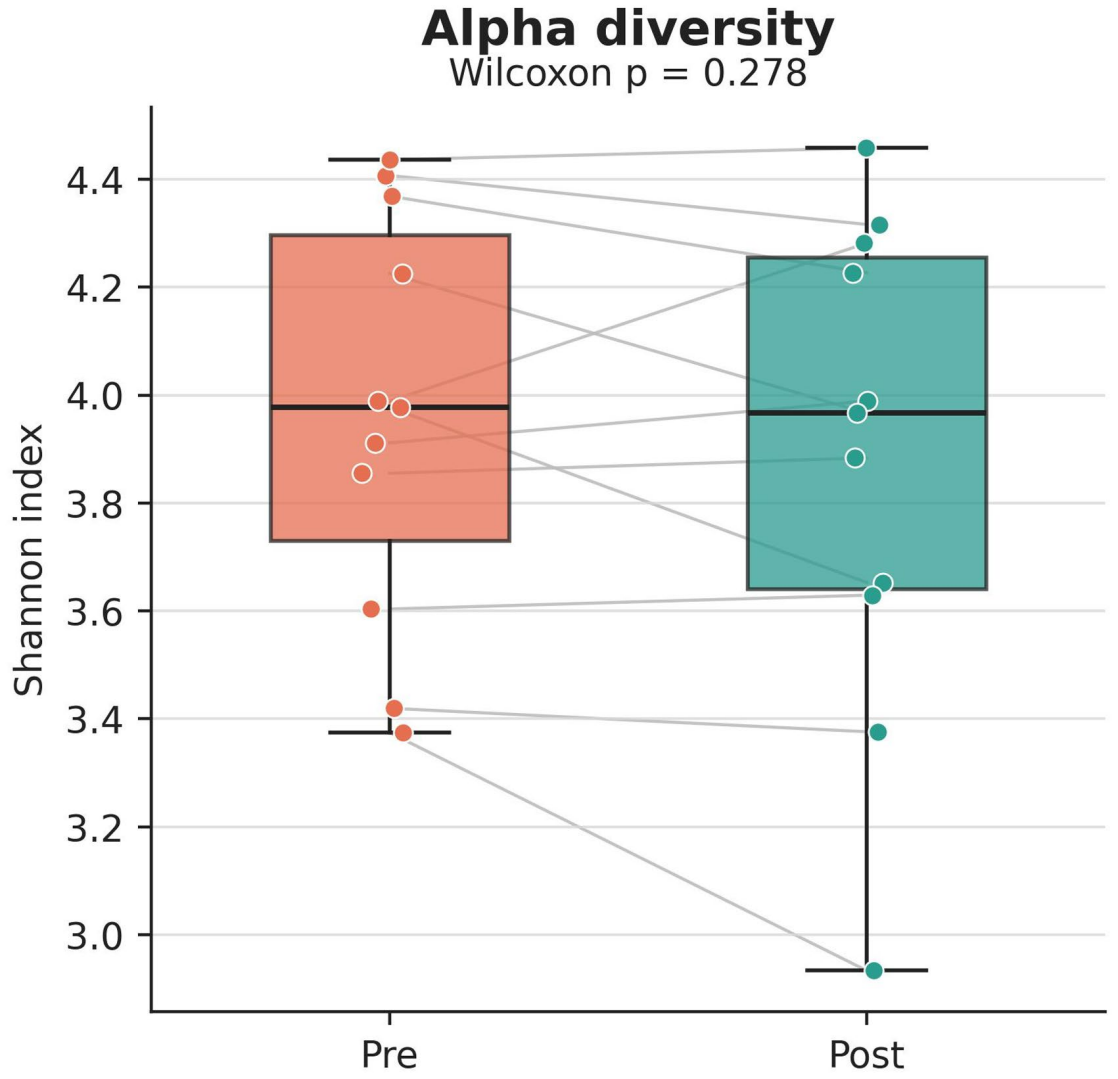
Shannon alpha diversity before and after the 8-week pre-competition training period in the simulated data package. Paired distributions are shown for pre- and post-training samples; no significant pre-post difference was observed (paired Wilcoxon signed-rank test, *p* = 0.278).

Shannon alpha diversity was used to summarize within-sample diversity before and after the 8-week pre-competition training. The re-plotted paired distributions in Figure 1, based on the simulated data package, did not suggest a clear pre-post difference, and the paired Wilcoxon signed-rank test was not significant (*p* = 0.278).

#### 3.1.2 Community structure before and after the pre-competition training

**Figure 2:**
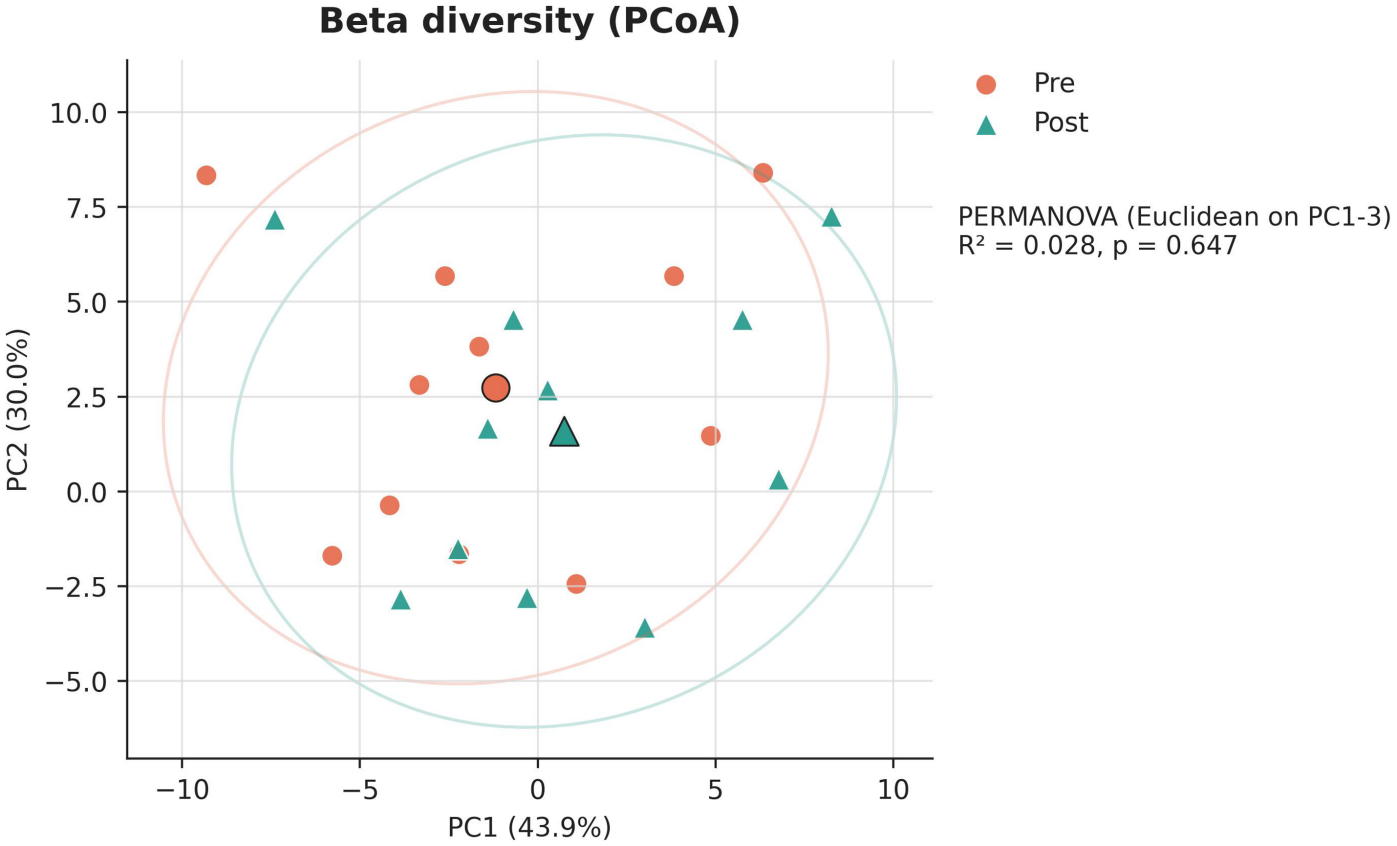
Principal coordinates analysis (PCoA) of gut microbiota beta diversity before and after the 8-week pre-competition training period in the simulated data package. PC1 and PC2 explained 43.9% and 30.0% of the displayed variance, respectively. Pre- and post-training samples overlapped substantially, and exploratory PERMANOVA on the reconstructed coordinate set was not significant (R^2^ = 0.028, *p* = 0.647).

A two-dimensional PCoA representation of the simulated gut microbiota coordinates was used to visualize beta diversity. In the plotted coordinate set, PC1 and PC2 accounted for 43.9 and 30.0% of the displayed variance, respectively. Pre- and post-training samples still overlapped substantially. Exploratory PERMANOVA on the reconstructed coordinate set was not statistically significant (R^2^ = 0.028, *p* = 0.647), supporting a cautious interpretation of overall community separation (Figure 2).

#### 3.1.3 Taxa showing exploratory pre-post differences

**Figure 3:**
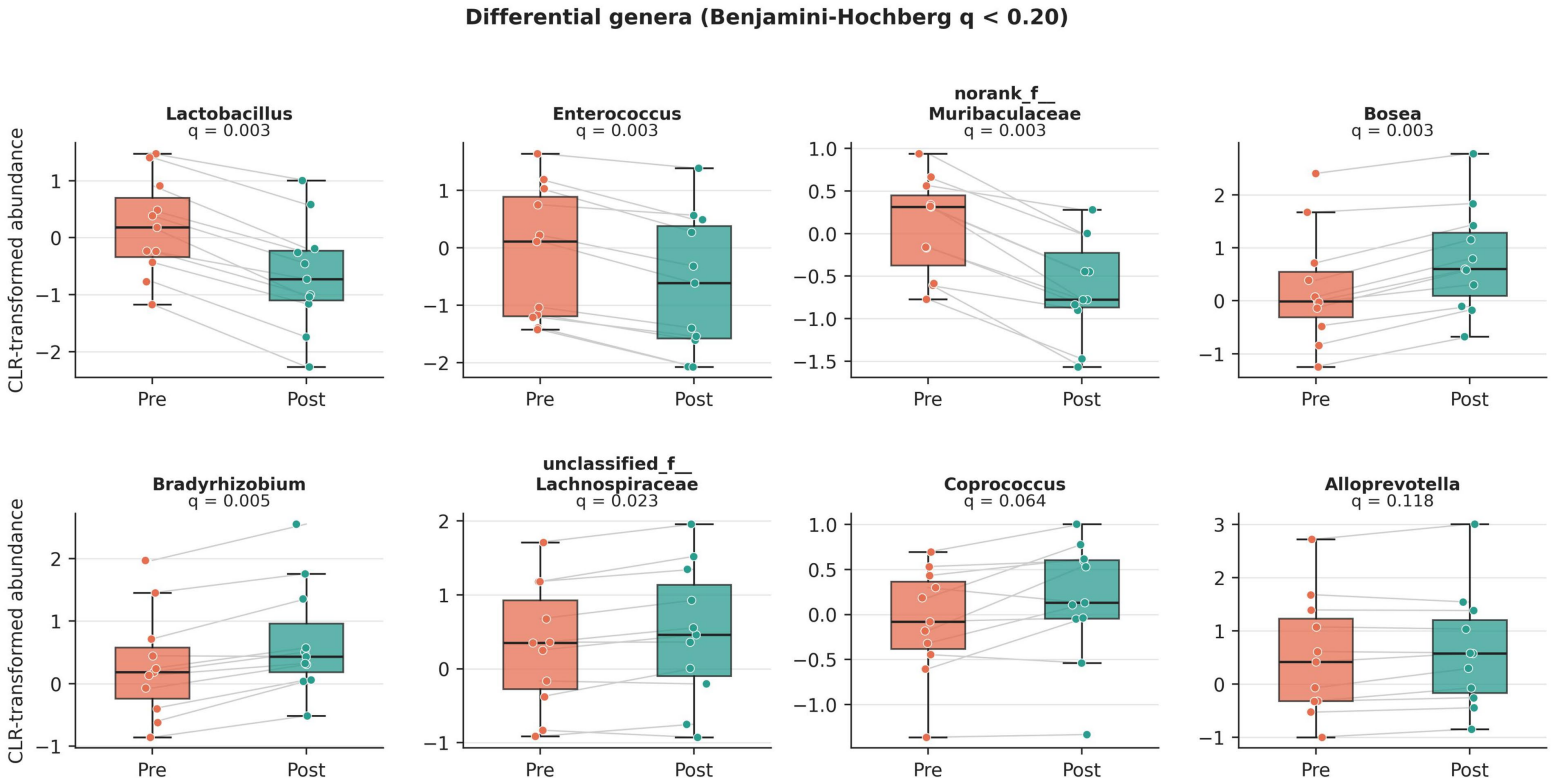
Exploratory pre-post differences in selected taxa reconstructed from the simulated data package. Benjamini-Hochberg q-values are shown for the selected reconstructed taxa.

The re-plotted exploratory differential-abundance summary in Figure 3 highlights the strongest reconstructed pre-post shifts among selected taxa in the simulated data package. The strongest reported FDR-supported signals were observed for *Lactobacillus*, *Enterococcus*, *norank_f__Muribaculaceae*, and *Bosea* (all q = 0.003), followed by *Bradyrhizobium* (q = 0.005) and *unclassified_f__Lachnospiraceae* (q = 0.023), whereas *Coprococcus* (q = 0.064) and *Alloprevotella* (q = 0.118) showed weaker or borderline signals. These results should be interpreted as exploratory candidate signals rather than definitive taxonomic effects.

### 3.2 Competition anxiety during the pre-competition training

**Figure 4:**
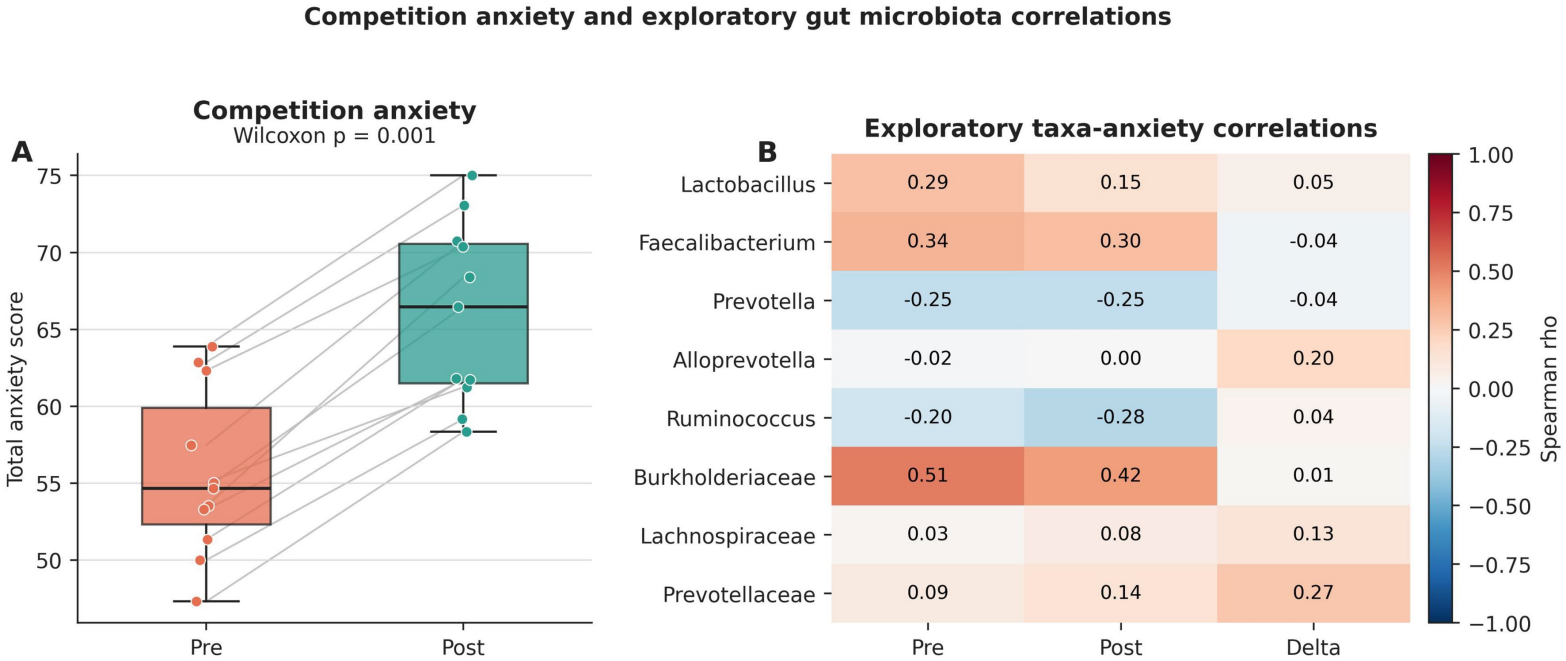
CSAI-2-derived competition anxiety-related responses before and after the 8-week training period **(A)** and a schematic heatmap of selected exploratory correlations between gut microbiota taxa and CSAI-2 subscale scores **(B)**.

According to the archived CSAI-2 analysis, cognitive anxiety and somatic anxiety were higher after the 8-week preparation period than before the start of training, whereas state self-confidence did not differ significantly between the two time points. These findings suggest that the pre-competition period was accompanied primarily by an increase in anxiety-related responses rather than by a clear change in perceived self-confidence (Figure 4).

### 3.3 Exploratory associations between gut microbiota and competition anxiety

Spearman correlation analysis was used to explore associations between gut microbiota taxa and CSAI-2 subscale scores. Table 3 reproduces the nominal exploratory associations reported for the archived questionnaire analysis. In the reported subset, Burkholderiaceae and Lachnospiraceae were associated with cognitive anxiety; Lactobacillaceae and Lactobacillus were associated with somatic anxiety; and Faecalibacterium, Prevotellaceae, Prevotella, Alloprevotella, and Ruminococcus were associated with state self-confidence.

## 4 Discussion

### 4.1 Exploratory gut microbiota changes during the pre-competition training

Regular exercise is a recognized modulator of the gut microbiota, but its effects are not uniform across populations, training modes, or microbiome outcomes. In the present study, 8 weeks of pre-competition training did not produce significant changes in alpha diversity or beta diversity, whereas several taxa differed significantly in relative abundance between the pre-training and post-training time points. This pattern suggests that the overall microbial ecosystem remained relatively stable during the training camp, while specific taxa were responsive to the combined effects of training load, competition preparation, dietary regularity, and closed-camp living conditions. Recent human intervention studies support this interpretation, showing that exercise may induce modest or inconsistent shifts in community-level diversity while still altering selected microbial taxa and functional profiles (Martinez-Tellez et al., 2025; Agyin-Birikorang et al., 2025; Mason et al., 2022).

More specifically, Lactobacillaceae/Lactobacillus, Enterococcaceae/Enterococcus, and Atopobiaceae increased after the pre-competition training, whereas Muribaculaceae, Beijerinckiaceae, Xanthobacteraceae, Saccharimonadaceae, Coprococcus, unclassified_f__Lachnospiraceae, norank_f__Muribaculaceae, Bosea, and Bradyrhizobium decreased. These taxonomic changes should be interpreted cautiously. In athlete cohorts, microbiome adaptation is often more apparent at the compositional or metabolic level than at the level of global diversity, and the biological relevance of many low-abundance or environmentally associated taxa remains uncertain (Fontana et al., 2023). Among the taxa identified here, Lactobacillus is of particular interest because recent clinical and translational studies suggest that lactobacilli can influence stress reactivity, sleep-related symptoms, and mood-related outcomes through microbiota-gut-brain signaling (Makela et al., 2023). By contrast, the functional meaning of changes in taxa such as Bosea or Bradyrhizobium in athletes is currently unclear, and these signals should not be overinterpreted as uniformly beneficial or harmful. Overall, our findings are more consistent with selective microbial remodeling during the preparation period than with a generalized increase in microbial diversity. Future studies using shotgun metagenomics, targeted metabolomics, and tighter dietary control are needed to determine whether these shifts reflect training adaptation, competition-related psychological stress, or other environmental exposures (Fu et al., 2024).

### 4.2 Competition anxiety during the pre-competition training

In the present cohort, the archived CSAI-2 analysis indicated that cognitive anxiety and somatic anxiety were higher after the preparatory phase, suggesting that the pre-competition period imposed a substantial psychophysiological burden on the athletes. This pattern is consistent with recent evidence showing that competition-related situational cues in taekwondo increase anxiety and arousal, and that stress responses intensify as athletes approach more decisive competition stages (Maloney et al., 2022; Paludo et al., 2022). The mechanisms are likely multifactorial: intensified training load, greater tactical and performance expectations, and the combined pressure of sport and non-sport demands can all amplify perceived stress (Drole et al., 2025). Sleep may be one pathway through which these pressures are translated into poorer psychological states. Although the present dataset included sleep duration rather than detailed sleep phenotyping, sleep remains a plausible potential confounder because sleep difficulty is common during pre-meet camps and major competitions and may be exacerbated by injury status and high-intensity training or match loads (Mason et al., 2022; Apinis-Deshaies et al., 2023). From a gut-brain-axis perspective, these stressors are biologically plausible modulators of the gut microbiota. In our data, alpha diversity remained stable and beta-diversity separation was limited, whereas several genera changed across the preparation period, including decreases in Lactobacillus, Enterococcus, and norank_f__Muribaculaceae and increases in Bosea and Bradyrhizobium, suggesting that short-term competitive stress may be expressed more readily at the taxon-specific level than at the level of overall community diversity. This interpretation is consistent with recent evidence that athletes exhibit distinctive gut microbial and metabolic signatures (Fontana et al., 2023), and with emerging data linking pre-competition anxiety to altered microbial and metabolomic profiles in wrestlers (Fu et al., 2024).

### 4.3 Exploratory associations between gut microbiota and competition anxiety

The gut microbiota may influence emotional regulation through immune, endocrine, neural, and metabolic pathways, while psychological stress can in turn reshape the intestinal microbial environment. Experimental work has demonstrated that gut-derived microbial metabolites can alter myelination, brain activity, and anxiety-like behavior, providing mechanistic support for the microbiota-gut-brain axis in affective regulation (Needham et al., 2022). More recently, fecal microbiota transplantation from patients with social anxiety disorder was shown to increase social fear behavior in recipient mice, further supporting a causal contribution of the gut microbiome to anxiety-related phenotypes (Ritz et al., 2024). Human genetic evidence is also beginning to converge with these observations; a recent bidirectional Mendelian randomization study reported potential causal links between specific gut microbial taxa and anxiety disorders (Li et al., 2024). In the sports context, a recent study in wrestlers likewise showed that pre-competition anxiety was associated with distinct gut microbiota and metabolite profiles, supporting the view that microbiome variation may be relevant to psychological readiness in competitive settings (Fu et al., 2024).

Against this background, our correlation analysis suggests that competition anxiety-related responses in taekwondo athletes may be associated with a distinct microbial signature. Taxa that were positively associated with anxiety, including Burkholderiaceae, Ruminococcus, Prevotellaceae, Prevotella, and Alloprevotella, may reflect a stress-related microbial pattern, although the direction and magnitude of these associations are likely to be context dependent. Recent human microbiome studies of internalizing disorders have emphasized that anxiety- and depression-related signals are usually distributed across microbial networks rather than attributable to a single genus, and that some associations differ across cohorts, clinical phenotypes, and medication status (Brushett et al., 2023). Therefore, the present findings should be regarded as exploratory ecological associations rather than evidence of direct causation.

By contrast, taxa that were negatively associated with competition anxiety-related outcomes, including Lachnospiraceae, Lactobacillaceae/Lactobacillus, and Faecalibacterium, are broadly consistent with a potentially protective microbial profile. Lachnospiraceae and Faecalibacterium include major short-chain fatty acid-producing taxa that are relevant to intestinal barrier integrity, immune homeostasis, and neuroactive metabolite production. In recent human and intervention studies, enrichment of Faecalibacterium- and Roseburia-related profiles has been associated with improved mood-related outcomes, whereas depletion of butyrate-producing bacteria has been repeatedly linked to internalizing symptoms (Jiang et al., 2023). Likewise, controlled psychobiotic trials indicate that Lactobacillus-related interventions can modulate state anxiety, stress reactivity, and sleep-associated complaints in psychologically stressed populations, although effect sizes remain modest and are strain specific (Makela et al., 2023). Taken together, our data support the hypothesis that gut microbiota composition is associated with competition anxiety during the preparation period. However, because of the small sample size, the cross-sectional nature of the correlation analysis, and the absence of metagenomic or metabolomic validation in this cohort, these observations should be interpreted as hypothesis-generating. Larger longitudinal studies are required to determine whether the identified taxa can serve as reproducible biomarkers or intervention targets for psychological management in high-performance athletes.

### 4.4 Strengths and limitations

A major strength of this study is that it evaluated gut microbiota changes and competition anxiety in high-performance taekwondo athletes within a real-world pre-competition training context using a paired pre- and post-training design. Several limitations should be acknowledged. First, the small sample size and the absence of a control group limited statistical power and precluded causal inference. Second, although the cohort included both male and female athletes, sex-specific effects could not be examined reliably. Third, while sleep duration was recorded, more detailed sleep-related measures were unavailable, and other potential confounders, including dietary intake, injury status, and day-to-day training load, were not fully modeled. Fourth, the microbiome analysis was based primarily on 16S rRNA sequencing without direct metabolomic validation, which limited functional interpretation. Finally, the lack of intermediate sampling points prevented assessment of the temporal dynamics of microbiota and anxiety changes during the pre-competition training. Despite these limitations, the study provides preliminary insight into the relationship between gut microbiota and competition anxiety in an understudied population of high-level athletes.

## 5 Conclusion

In conclusion, this exploratory pilot study suggests that the pre-competition training period in high-performance taekwondo athletes was associated with changes in selected gut microbial taxa and with higher competition anxiety-related responses. Although no clear pre- to post-training differences were observed in overall alpha or beta diversity, several specific taxa showed differential abundance over the training period. In addition, exploratory correlation analyses indicated that multiple gut microbial taxa were associated with CSAI-2 subscale scores, supporting a potential link between gut microbiota composition and competition anxiety during the preparation period. However, given the small sample size, the absence of a control group, the mixed-sex cohort, and the exploratory nature of the analyses.

## Data Availability

The original contributions presented in the study are included in the article/supplementary material, further inquiries can be directed to the corresponding author.
